# Evaluation of micronuclei and antioxidant status in hospital radiation workers occupationally exposed to low-dose ionizing radiation

**DOI:** 10.1186/s12913-023-09516-2

**Published:** 2023-05-25

**Authors:** S. N. Mousavikia, M. T. Bahreyni Toossi, S. Khademi, M. Soukhtanloo, H. Azimian

**Affiliations:** 1 Medical Physics Research Center, Mashhad University of Medical Sciences, Mashhad, Iran; 2 Department of Radiology Technology, School of Paramedical Sciences, Mashhad University of Medical Sciences, Mashhad, Iran; 3 Department of Biochemistry, School of Medicine, Mashhad University of Medical Sciences, Mashhad, Iran

**Keywords:** Ionizing radiation, Adaptive-response, Micronuclei, Antioxidants, Oxidative stress

## Abstract

**Purpose:**

There is scientific evidence that ionizing radiation (IR) can be responsible for various health hazards that are one of the concerns in occupational exposure. This study was performed to evaluate DNA damage and antioxidant status in hospital workers who are occupationally exposed to low doses of IR.

**Materials and methods:**

In this study, twenty occupationally exposed to low doses of IR (CT and angiography) comprising with control groups which matched them. In order to investigate the effects of chronic irradiation of radiation workers, Micronuclei (MN) frequency and the antioxidant activity of Superoxide Dismutase (SOD), Catalase (CAT) and Total Antioxidant Capacity (TAC) were measured. Then, to check adaptation against high challenge dose, the samples (in all groups) were irradiated in vitro and MN frequency was compared. Finally, to investigated the effect of the high dose after the acute and chronic low dose of ionizing radiation, MN frequency was compared in two groups (the control group that was to in-vitro irradiated (acute low dose + high dose) and radiation workers (chronic low dose + high dose)).

**Results:**

MN frequency in the occupationally exposed group (*n* = 30) increased significantly when compared to the control group (*p*-value < 0.0001). However, chronic irradiation of radiation workers could not lead to an adaptive Sresponse, while acute low-doses could produce this effect (*p*-value ˂ 0.05). In addition, the activity levels of antioxidant enzymes SOD, CAT, and TAC were not statistically different between the radiation workers and the control group (*p*-value > 0.05).

**Conclusions:**

We observed that exposure to low doses of IR leads to increased cytogenetic damage, could not cause an adaptive-response, and improve antioxidant capacity in radiation workers. Controlling healthcare workers' exposure is the first step to improving the health of hospital workers and the quality of patient care, thus decreasing human and economic costs.

## Introduction

Ionizing radiation (IR) plays an important role in the modern world. Humans are constantly exposed to these rays through the environment, occupation, medical use, or other sources [1]. We know that IR is effective in treating and diagnosing various diseases for patients, but the effects of their occupational exposure on medical staff such as cataracts, cardiovascular disease and of course cancer cannot be ignored. Although the dose received by most hospital staff who are exposed to low doses of ionizing radiation (LDIR) lied within the specified range of The International Commission on Radiological Protection (ICRP), but in recent decades the use of high-dose techniques has raised concerns. A personal dosimeter that is routinely used may underestimate the actual exposure, not only for the detecting threshold of dosimeters but maybe it is in the wrong place. Note that the occupational hazards of these individuals are not limited to the time they are on duty, but their effects will increase with more exposure to IR, and according to the available evidence, may affect future generations [2, 3]. Although the effects of high doses of ionizing radiation (HDIR) are well known, the equivocations of LDIR have still remained ambiguous [4]. Biological responses to LDIR depend on various physical factors. The first and most important factor is the total absorbed dose and nature of radiation exposure, i.e., acute or chronic irradiation [5]. Other factors include the distribution of radiation sources and dimensions of biological targets [6]. We know that IR causes single-strand and double-strand DNA breaks and genomic instabilities [7]. These lesions are usually diagnosed and repaired quickly by cellular mechanisms, but few remain and are seen later in the cell cycle. According to the studies after radiation to adapt and maintain the survival of the cells, various pathways are activated in different time [8]. For example, after a few hours of radiation, reactive oxygen species (ROS) scavengers and signaling pathways leading to apoptosis are activated. Within a few days, DNA repair pathways and after several weeks, immune responses appear [9]. On the other hand, LDIR can reduce the genomic damage to human lymphocytes caused by subsequent high doses. The phenomenon is an example of an "adaptive-response" which is often evoked as possible mechanisms to stimulate specific protective functions [10].

IR interacts directly with specific molecules in the cell, including water [11]. In this interaction, most of the IR energy is spent expelling electrons from the water molecule. The product of such a collision is ROS including hydroxyl radicals (OH°), which are highly reactive and, if formed near biological molecules, have the potential to cause immediate oxidative damage [12]. The occurrence of chain reactions and the production of destructive free radicals leads to a series of damages, generally referred to as oxidative stress [13]. Nevertheless, antioxidants are an important defense system to counteract the effects of this oxidative stress which contribute to removing ROS produced both directly or indirectly [14].

IR can cause oxidative stress and subsequently produce ROS and release free radicals. Its effects in the DNA damage—the indirect effect—and even changes in mitochondrial physiology -as the center of oxidative metabolism-. Finally, it can lead to increase genomic instability in irradiated cells [15, 16]. One way to protect against ROS is through antioxidant molecules such as glutathione (GSH) as well as antioxidant defense enzymes such as catalase (CAT) and superoxide dismutase (SOD). There is usually a balance between ROS and antioxidants, but an imbalance can result from a disease or prolonged exposure to IR, which can lead to oxidative stress. Chronic oxidative stress is itself a cause of DNA damage leading to many diseases such as cancer [17]. Various hypotheses have been proposed to describe the adaptive-response mechanisms of LDIR. The most important is to stimulate the immune system, accelerate DNA damages detection and repair, and increase antioxidant levels [18].

However, concern about occupational exposures increased with the publication of study results in the first half of the past century. Therefore, creating restrictions to maintain health and prevent adverse effects was a serious matter [19]. In this regard, ICRP recommends a dose limit of 50 mSv in any one year and 20 mSv averaged over defined periods of 5 years [20]. One of the occupational groups that are most consistently exposed to LDIR is hospital workers [21]. So far, in studies, the effects of this chronic radiation have been measured using various methods in the changes of different factors. Such as examining chromosomal abnormalities (dicentric, ring, translocation), cytokinesis block micronucleus (CBMN), premature chromosome condensation (PCC), comet assays, and as well as hematological and biochemical parameters [22, 23]. Each of these methods has strengths and weaknesses. Among them, micronuclei assay is a reliable and sensitive method, especially for low doses [24].

The present study was performed to analyze the cytogenetic damage associated with IR as well as to evaluate the incidence of adaptive-response using a micronucleus test plus measurement of antioxidant levels in hospital workers exposed to occupational radiation from LDIR. Their results are then compared with the control group findings.

## Materials and methods

### Selection of subjects

Participants in this study included 30 volunteers recruited with informed consent. Studies have shown that age, gender, smoking habits, and residence can affect the results. To minimize these factors, all volunteers in this study were male and minority smokers. They were all asked to fill in a questionnaire including questions about lifestyle, work experience, and medication. The exposed participants (*N* = 20) included radiation workers in CT scan and angiography working in Imam Reza Hospital in Mashhad. The control group (*N* = 10) matched the exposed groups regarding gender, age, and smoking status. The characteristics of each of the 30 volunteers are summarized in Table 1. Calibration procedures of all exposure systems was done according to regional regulations and approved by National Radiation Protection Department (NRPD) of Nuclear Regulatory Authority (INRA).

**Table 1:** The demographic characteristics of exposed and control groups

**participants**	**Control group**	**Medical exposure**	
		**CT**	**Angiography**
Number of volunteers	10	10	10
Age (year) Mean ± SD	38.1 ± 9.06	36.7 ± 5.73	39.5 ± 8.97
Duration of employment (years) Mean ± SD	-	15.7 ± 4.19	17.7 ± 7.11
Smoking Habit			
Yes:NO	2: 8	2: 8	2: 8

### Irradiation of sample

**Fig. 1:**
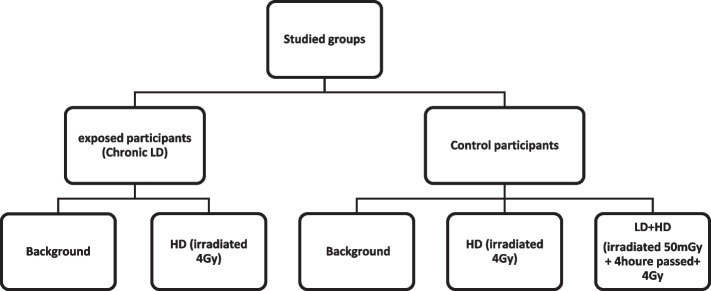
Schematic of the studied groups and their related codes

The blood samples of the exposed participants were divided into two sub-groups, background damage assessment, and the other by applying a dose of 4 Gy to evaluate adaptive-response induction (HD). Also, for the control group, the samples were divided into three sub-groups, to investigate the background damage, irradiated at a dose of 50 mGy and after 4 h at a dose of 4 Gy (LD + HD), and the last flask was applied only 4 Gy dose of IR (HD) as shown schematically in Fig. 1. Irradiation with 6 MV accelerators was performed in the field of 20 × 20, SSD = 100 cm at a 180-degree gantry angle. The dose rate used to deliver the Low Dose (50 mGy) was considered to be 50 cGy/min, and for delivering the High dose (4 Gy) dose rate was 200 cGy/min.

### Blood sampling

For each participant, nearly 0.5 mL of the peripheral blood sample was taken in heparinized vacuum tubes (Becton Dickinson) to perform the CBMN test. Approximately, 2 mL of the blood sample was poured into the clot tube and the separated plasma was stored at -80 °C to analyze the oxidative plus antioxidant levels.

### Cytokinesis-Block Micronucleus (CBMN) assay

The CBMN assay was performed according to Fenech and Morley, albeit with minor modifications to achieve the best efficiency [25–27]. Half a mL of peripheral whole blood was added to RPMI 1640 medium (Gibco) containing antibiotics (penicillin 100 IU/mL and streptomycin 100 μg/mL (Sigma), 20% fetal bovine serum (Gibco), and 1% phytohemagglutinin, and incubated at 37 °C and 5% CO2 for 72 h. After 44 h from culture initiation, cytochalasin-B (Sigma) was added at a final concentration of 5 μg/mL. Lymphocytes in peripheral blood were fixed in cold and fresh methanol/acetic acid (6:1), then air-dried and stained with 7% Giemsa solution. Two parallel cultures were determined per subject. So, in a total of 1000 binucleated (BN) lymphocytes (The two nuclei in a BN cell should have intact nuclear membranes and be situated within the same cytoplasmic boundary, approximately equal in size and staining intensity), the frequencies of MN scored (The boundary should be distinguishable, have the same staining intensity as the main nuclei but smaller and not overlap with them) were analyzed.

### Measurements of antioxidant level

After frozen plasma samples were reached room temperature, samples were subjected to the following determinations: SOD activity via the madesh colorimetric method, CAT activity through formaldehyde colorimetry, and TAC upon oxidation based on ferryl myoglobin radicals. SOD, CAT, and TAC levels were quantitated by using ELISA (570 nm for SOD, 540 nm for CAT, and 412 nm for TAC) in accordance with the kit instructions (Teb Pazhouhan Razi, Tehran, Iran).

### Statistical analysis

In this study, GraphPad Prism software version 8.0.2 was used to analyze the data. Firstly, the normal distribution of data in all groups was investigated. Then, for statistical analysis of the results between two independent groups, t-test and U test (Mann–Whitney) were employed for normal and abnormal distribution, respectively. Also, one-way ANOVA test was used for statistical analysis of the results between several groups with normal distribution.

## Results

### The demographic characteristics of participants

The demographic characteristics of all participants in the study are summarized in Table 1.

It should be noted that none of the participants in the study exceeded the occupational dose limit of 20 mSv / year. There was no statistically significant difference between the ages of groups. Also, due to hormonal changes in women and the possibility of gender affecting the MN frequency, only male participants were selected. Since many substances in cigarette smoke are genotoxic, and therefore, cytogenetic damage seems to be a suitable biomarker to determine the effect of smoking [28]. A smaller number of smoking participants were recruited. These differences were not statistically significant between groups. And because many studies, a significant relationship between MN frequency and increasing age has been reported [29]. Therefore, the volunteers selected that there is no statistically significant difference between their average age in all groups.

### Oxidative stress

**Fig. 2:**
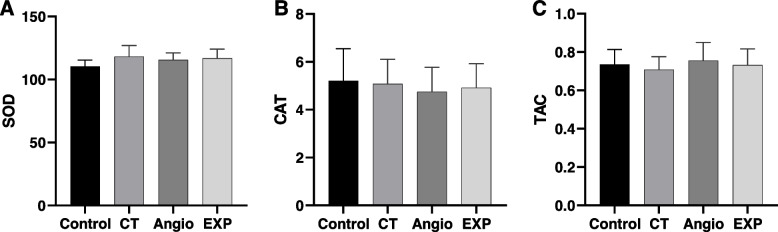
**A** Mean SOD activity obtained in exposed group (EXP = angiography + CT), CT, angiography and control, (**B**) Mean CAT activity obtained in exposed group (EXP = angiography + CT), CT, angiography and control, (**C**) Mean TAC. Error bars show standard deviation

To evaluate the possibility of increasing antioxidant capacity in radiation workers,the levels of antioxidants (is summarized in Table 2) among the participants were compared between the two groups of exposed and controls (Fig. 2). The *P*-value obtained from statistical analysis using the ANOVA test showed more than 0.05 in the comparison of groups. So there is no significant difference between any of the groups at the level of SOD (Fig. 2A), CAT (Fig. 2B), and TAC (Fig. 2C) (*p*-value > 0.05).

**Table 2:** The levels of antioxidants (mean ± SD) for all groups

	Control	CT	Angiography	Exp(CT + Angio)
SOD	110.4 ± 5.043	118.1 ± 8.814	115.5 ± 5.565	116.8 ± 7.299
CAT	5.2130 ± 1.339	5.0795 ± 1.026	4.669 ± 1.112	4.874 ± 1.062
TAC	0.7357 ± 0.078	0.7087 ± 0.067	0.7553 ± 0.095	0.7320 ± 0.084

### Cytogenetic analysis in exposed and control groups

**Fig. 3:**
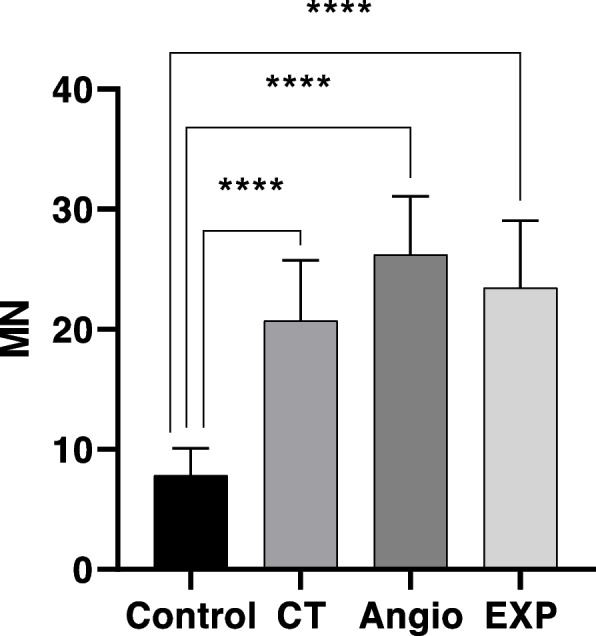
Mean MN obtained in the exposed group (EXP = angiography + CT), CT, angiography, and control. Error bars show standard deviation

**Fig. 4:**
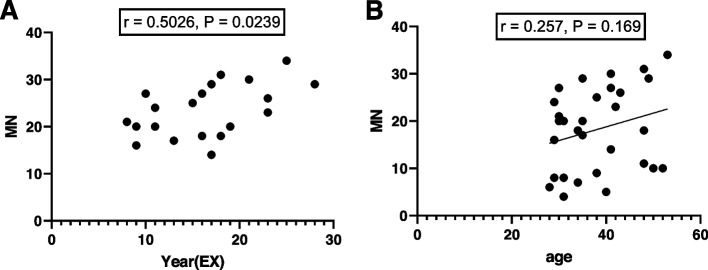
**A** The mean frequencies of micronuclei in medical radiation workers based on working duration, (**B**) The mean frequencies of micronuclei in medical radiation workers based on age

**Fig. 5:**
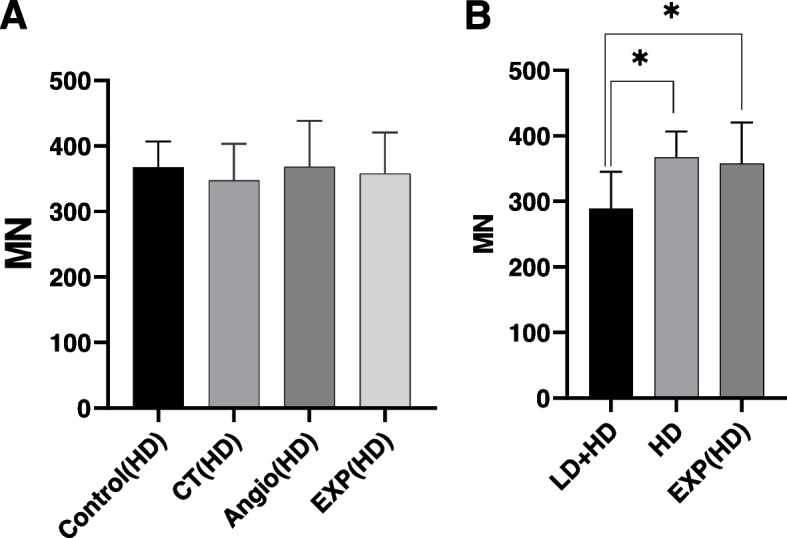
**A** Mean MN obtained exposed group (EXP = angiography + CT), CT, angiography and control after 4 Gy (HD) irradiation. **B** Mean MN was obtained in two groups of LD + HD (first 50 mGy irradiation, 4 h elapsed and 4 Gy irradiation) and HD (4 Gy irradiation only) of the control group and exposed group (EXP = angiography + CT) after 4 Gy (HD) irradiation to evaluate the adaptive-response due to acute low dose. Error bars show standard deviation

The MN frequency (mean ± SD) for all groups is summarized in Table 3. The MN frequency in the exposure group was significantly higher than in the control group (*p*-value < 0.0001) (Fig. 3). Also, there was a significant correlation between MN and years of employment among radiation workers (*r* = 0.5026, *P* = 0.0239) (Fig. 4A). However, there was no significant correlation between MN and age in any of the groups within this age range (*r* = 0.257, *P* = 0.169) (Fig. 4B). There was no statistically significant difference either in MN frequency between smokers and non-smokers in both groups (*p*-value = 0.479). The MN frequency evaluation did not confirm the occurrence of the adaptive-response in chronically exposed group (Fig. 5A). On the other hand, incidence of adaptive-response has been obvious in acute LD group (Fig. 5B). In this comparison, it was assumed that radiation workers received chronically LD during their years of employment.

**Table 3:** The MN frequency (mean ± SD) for all groups

	Control	CT	Angiography	Exp(CT + Angio)
Background	7.8 ± 2.3	20.7 ± 5.03	26.20 ± 4.8	23.45 ± 5.5
LD + HD	289.4 ± 55.8	-	-	-
HD	367.1 ± 39.8	347.2 ± 55.9	368.2 ± 70.3	357.7 ± 62.7

## Discussion

In this study, two assays (biochemical to evaluate oxidative stress and micronucleus to assess background damage and adaptive-response) were used to evaluate the effects of exposure to LDIR on radiation workers. In the present study, the antioxidant levels of SOD, CAT, and TAC were measured in medical radiation workers and compared with the control group. SODs are part of the enzyme's defense system against oxidative stress by converting the superoxide radical anion to ${H}_{2}{O}_{2}$ [30]. Our results revealed that the level of antioxidant SOD in radiation workers was not significantly different compared to the control group (Fig. 2A). Kumar et al. reported that the level of SOD in exposures was significantly lower than in the control group [31], and paradoxically some researchers even found a significant increase in SOD levels in radiation workers [32, 33]. CAT is an antioxidant enzyme that converts ${H}_{2}{O}_{2}$ to water. This enzyme is expressed in most cells, organs, tissues, and of course, at high concentrations in the liver and red blood cells [34]. In this study, the level of this enzyme in blood serum was measured, and no statistically significant difference was observed between the two groups of exposures and control (Fig. 2B). This result is in agreement with some previous studies [10, 32]. However, there are some contradictory results [35–38]. Finally, TAC was measured to calculate all antioxidants present in the biological sample [39]. In the present study, there was no statistically significant difference in the level of TAC between the radiation workers and control groups (Fig. 2C). This contradicts the report of Kumar et al., who reported that the TAC in radiation workers was significantly higher than in the control group [31].

It was previously reported that LDIR exposure can accelerate cellular aging by increasing ROS activity [40]. Evidence for ROS involvement in mechanisms is mainly associated with an external physical or chemical disturbance, of which radiation may be a major factor [38]. Meanwhile, strong antioxidant enzymes play a vital role in oxidative stress responses [41]. This is because there is a probability of a slight increase in oxidative stress in response to chronic occupational exposure to LDIR [42]. However, in this study it was observed that there was no significant difference in the levels of SOD, CAT, and TAC antioxidants between radiation workers and control group. Thus, these results do not support the theory of Hormesis.

Cytokinesis- block micronucleus assay has been used as a quantitative indicator of chromosomal damage both in-vitro and in-vivo in many studies [43, 44]. The results of this study indicated that the average frequency of MN in medical exposures was significantly higher than in the control group which is consistent with the results of other studies [31, 35, 37, 45–54]. However, some studies have not reported an increase in the frequency of MN [55–57]. This may be due to confounding factors such as gender or the dose received by the radiation. Interestingly, there was a significant relationship between the MN frequency and years of employment (exposure to IR), which was positive (*r* = 0.5026, *P* = 0.0239) (Fig. 4A). As the years of working with IR increased, so did the frequency of MN, with this result concurring with many studies [36, 37, 47–49, 51, 52, 54–57]. Some studies have reported an increase in the frequency of MN as a function of age in medical exposure [37, 51]. However, in this study, no relationship was observed between age and MN frequency, which is consistent with the reports of some studies [45, 52]. Naturally, increasing the years of employment is accompanied by increasing age, and separating the effect of these two factors requires more research.

The results of studies examining the relationship between smoking habits and MN frequency are highly controversial. Several studies have reported a significant association between smoking and increased MN frequency in hospital workers and control radiologists [54, 56]. In others, no significant relationship was found in either hospital workers or control radiographs, which is similar to the results of the present study [31, 36, 52].

Adaptive-response is a term used to describe an organism's reaction to IR exposure or chemicals to minimize subsequent damages by activating various mechanisms such as gene expression or the synthesis of specific proteins [58–60]. To date, various studies have been performed on cells, plants, animals, and humans using different methods to examine the adaptive-response [61, 62]. However, studies on human blood lymphocytes have shown that this protective phenomenon does not occur equally in all individuals [63]. In other words, it does not occur as a general principle for all organisms and under different conditions. The genetic status of individuals in response to this phenomenon is important. Thus, it seems that the adaptive-response cannot be considered valid in the practical application according to the general regulations of radiation protection [64]. So far, dose–response curves have been plotted using different methods. These curves have shown that due to dose elevation, the frequency of cytogenetic damage will also increase [65].

However, some studies also emphasize that in some cases, by activating different signaling pathways at different doses, less damage may occur in high doses [66]. In this study, the damage after application of 4 Gy was examined using CBMN assay in two groups of medical radiation workers and control. Our results revealed that there was no statistically significant difference between the two groups after the application of the 4 Gy dose (Fig. 5A). This result was in agreement with the report of Jasik et al. [67]; They reported lack of adaptive-response by examining the level of chromosomal abnormalities, apoptosis, and MN between the two groups of radiation workers and control. In contrast, Barkinero et al. [68] examined chromosomal abnormalities in 12 radiologists and compared the results with eight subjects as a control group. They reported that after 2 Gy irradiation, the frequency of chromosomal abnormalities in radiologists was significantly lower than in the control group. Also, Gorabi et al., who used the micronucleus test in their study, reported that the frequency of MN during irradiation of 1 Gy and 2 Gy was significantly lower than in the control group [69]. In 2011, Rasso et al. reported a statistically significant difference between caspase-3 activity after 2 Gy between 10 cardiovascular staff and 10 control groups [10]. In another study that was conducted to investigate the adaptive response in people who are at a background radiation level higher than normal, they reported that the mean frequency of MN was significantly lower in elder (> 40 years) individuals from high-level natural radiation areas as compared to the young (≤ 40 years) individuals after 1 Gy and 2 Gy of challenging doses [70]. In the similar study, with proteomic approach was employed to study the response of human peripheral blood mononuclear cells. A total of 15 proteins were found to be statistically altered in individuals from high-level natural background radiation areas when compared to individuals from normal-level natural radiation areas. More importantly, when challenged with an invitro dose of 2 Gy, added to a sample with a high dose background, responded with an up-regulation of many protective pro-survival proteins such as protein disulfide- isomerase A1 (PDIA1), peroxiredoxin 6 (PRDX6) and glucose-regulated protein 78 kDa (GRP78). This indicates that human cells respond to LDIR through changes in the proteome to maintain adaptive homeostasis [71].

In this study, a comparison was made between two groups of controls, namely LD + HD (first 50 mGy irradiation, 4 h elapsed, and then 4 Gy irradiation) and HD (only a 4 Gy dose). Our results showed that the frequency of MN in the LD + HD group was significantly lower than in HD exposed to control group and radiation workers (Fig. 5B), It can suggest the adaptive-response induced by acute LD and not caused by chronic radiation. However, further studies are needed to confirm these results.

Studies examining the adaptive-response on human lymphocytes by applying HD following a LD in various ways have reported that damage could be mitigated by this protocol. Shelke et al. observed the occurrence of adaptive-response after a 2 Gy of gamma radiation, 4 h after receiving 0.1 Gy, by examining NHEJ repair pathway genes on peripheral blood samples from 20 healthy individuals [60]. In another study by Toprani et al., a significant reduction in DNA damage to the blood 5cells in 12 of the 20 samples was observed after 2 Gy gamma irradiation following a 0.1 Gy dose [72]. Since the dose rate is likely to be an important factor in creating the IR effects [73], accordingly, the occurrence of IR effects in low dose-rate (chronic radiation over the years) and high dose-rate (acute radiation) may be different. In this study, this issue was investigated by comparison in two groups. The results indicated that there was no significant difference between any of the HD groups of radiation workers and control (Fig. 5A). These results strongly rule out the adaptive-response induced by chronic low doses. Note that no study has investigated such an issue so far.

Undoubtedly, our research also has limitations and weaknesses. The biological response to DNA damage involves activating DNA repair and cell signaling pathways, which can ultimately affect cell cycle checkpoint arrest and/or apoptosis [74]. Of course, the way of response during long-term exposure to LDIR has not been clearly defined. Therefore, it seems that conducting studies on a wide range of radiation workers and examining different pathways of cell signaling and biomarkers involved in it will help greatly. In addition, follow-ups of radiation workers during their years of employment in response to the effects of factors such as age, sex, smoking, and even years of radiation exposure gave more definitive opinions. Thus, it seems interwoven processes occur at low dose and low dose rate of IR and further studies are required to clarify all aspects of the issues of hormesis and adaptive-response hypothesis.

## Conclusion

In this study, oxidative stress analysis was performed through measuring three levels of antioxidants including SOD, CAT, and TAC. Simultaneously, micronucleus frequencies were used to evaluate chronic exposure to LDIR on the health of radiation-workers.

We also discussed the occurrence of adaptive-response as one of the effects in the LDIR and investigated the occurrence of adaptive-response with an acute low dose and chronic low dose. Overall, our observations showed that chronic exposure to LDIR increases cytogenetic damage. In addition, it reduced neither the cytogenetic effects of subsequent high doses nor altered the antioxidant levels.

Therefore, to evaluate the genotoxic effects of chronic exposure to ionizing radiation in radiation workers and the more secure use of IR in medicine, in addition to the continuous monitoring of medical radiation workers by using personal dosimeters and periodic health examinations, biological markers are to be likewise, more comprehensive studies should be done.

## Data Availability

Data are available on request due to privacy or ethical restrictions. If someone wants to request the data of this study, should contact Dr. Hosein Azimian as the corresponding author.

## Abbreviations

IR: Ionizing Radiation
MN: Micronuclei
BN: Binucleated
SOD: Superoxide Dismutase
CAT: Catalase
TAC: Total Antioxidant Capacity
LDIR: Low Doses of Ionizing Radiation
HDIR: High Doses of Ionizing Radiation
ROS: Reactive Oxygen Species
GSH: Glutathione
CBMN: Cytokinesis Block Micronucleus
PCC: Premature Chromosome Condensation
ICRP: The International Commission on Radiological Protection
